# Zinc Chelation Reduces Hippocampal Neurogenesis after Pilocarpine-Induced Seizure

**DOI:** 10.1371/journal.pone.0048543

**Published:** 2012-10-31

**Authors:** Jin Hee Kim, Bong Geom Jang, Bo Young Choi, Lyo Min Kwon, Min Sohn, Hong Ki Song, Sang Won Suh

**Affiliations:** 1 Department of Physiology, Hallym University, College of Medicine, Chuncheon, Korea; 2 Inha University, Department of Nursing, Incheon, Korea; 3 Department of Neurology, College of Medicine, Hallym University, Chunchon, Korea; University G. D’Annunzio, Italy

## Abstract

Several studies have shown that epileptic seizures increase hippocampal neurogenesis in the adult. However, the mechanism underlying increased neurogenesis after seizures remains largely unknown. Neurogenesis occurs in the subgranular zone (SGZ) of the hippocampus in the adult brain, although an understanding of why it actively occurs in this region has remained elusive. A high level of vesicular zinc is localized in the presynaptic terminals of the SGZ. Previously, we demonstrated that a possible correlation may exist between synaptic zinc localization and high rates of neurogenesis in this area after hypoglycemia. Using a lithium-pilocarpine model, we tested our hypothesis that zinc plays a key role in modulating hippocampal neurogenesis after seizure. Then, we injected the zinc chelator, clioquinol (CQ, 30 mg/kg), into the intraperitoneal space to reduce brain zinc availability. Neuronal death was detected with Fluoro Jade-B and NeuN staining to determine whether CQ has neuroprotective effects after seizure. The total number of degenerating and live neurons was similar in vehicle and in CQ treated rats at 1 week after seizure. Neurogenesis was evaluated using BrdU, Ki67 and doublecortin (DCX) immunostaining 1 week after seizure. The number of BrdU, Ki67 and DCX positive cell was increased after seizure. However, the number of BrdU, Ki67 and DCX positive cells was significantly decreased by CQ treatment. Intracellular zinc chelator, N,N,N0,N-Tetrakis (2-pyridylmethyl) ethylenediamine (TPEN), also reduced seizure-induced neurogenesis in the hippocampus. The present study shows that zinc chelation does not prevent neurodegeneration but does reduce seizure-induced progenitor cell proliferation and neurogenesis. Therefore, this study suggests that zinc has an essential role for modulating hippocampal neurogenesis after seizure.

## Introduction

It is well known that seizure increases adult neurogenesis in the subgranular zones (SGZ) of dentate gyrus of hippocampus in both the rodent and human brain [1], [2], [3]. Newly-born dentate granule cells that arise as a result of seizure integrate into existing hippocampal circuitry and may provide network plasticity for hippocampus-dependent learning and memory. Therefore, it is important to study how neurons are born in response to epileptic seizure and functionally integrated into the existing neural networks. Several factors influencing the functional integration of new-born neurons seem to be excessive neuronal activity and pro-inflammatory signaling. Severe seizure induced a short-term increase in the proliferation of neural progenitors, but most of the new cells died at 4 weeks after insult. However, the exact mechanisms by which seizure regulates progenitor cell proliferation and neurogenesis are not well understood.

Our previous study demonstrated that hypoglycemic brain insult transiently increases the number of proliferating progenitor cells and immature neurons in the SGZ of rats, followed by a sustained decline of progenitor cell proliferation and immature neurons 4 weeks later [4]. The mechanism underlying the rise and decline of hippocampal progenitor cell proliferation after hypoglycemia is unclear. However, we have proposed that synaptic zinc release from mossy fiber terminals is a key factor in this process, i.e. massive release of synaptic zinc after hypoglycemia stimulates neurogenesis, but reduced zinc release or reduced amount of vesicular zinc decreased neurogenesis [5].

The divalent cation zinc is the second most abundant transition metal in the brain following iron. Chelatable zinc is highly localized in the synaptic vesicle of mossy fiber terminals of the dentate granule cell [6], [7]; sites where neurogenesis and neural migration are most active in the adult brain [8]. Zinc has long been recognized as a biologically essential element for brain physiology [9], [10], [11]. It is an essential component of more than 300 enzymes and thus involved in the regulation of a wide variety of cellular processes, including cell division and DNA synthesis [12]. Zinc also influences hormonal regulation of cell division, specifically, those cells regulated by insulin-like growth factor-I (IGF-I) [12] or nerve growth factor (NGF) [13]. Division and migration of cerebellar granular cells is reduced after severe zinc deficiency [14], [15]. Golub et al. showed that zinc deficiency impaired performance in short-term-memory tasks [16]. Thus, the evidence described above suggests that zinc is an essential element required in cell division, proliferation, migration and development, and further suggests that this element may play a critical role in neurogenesis and cognitive function.

The present study sought to determine the role of vesicular zinc in modulating hippocampal neurogenesis after pilocarpine-induced seizure by using a cell permeable zinc chelator, (5-chloro-7-iodo-8-hydroxyquinoline; clioquinol, CQ) to test the requirement for zinc on post-seizure neurogenesis.

**Figure 1:**
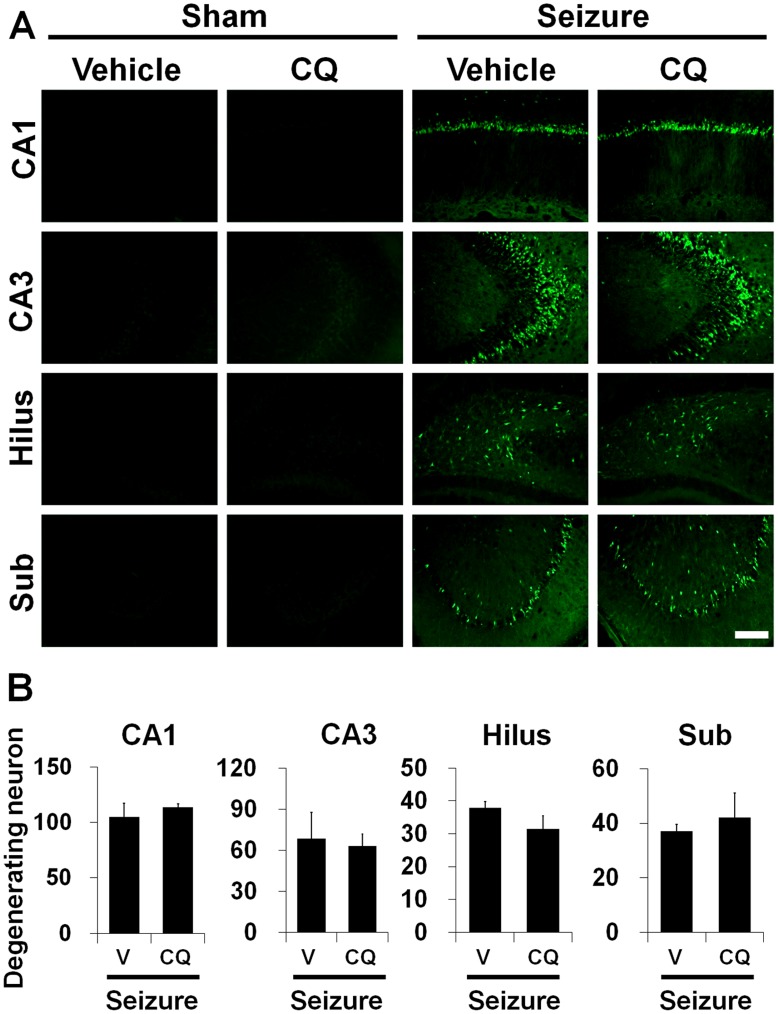
Seizure-induced hippocampal neuron death is not prevented by clioquinol. (A) Pilocarpine-induced seizure produced neuronal death in the hippocampal CA1, CA3, Hilus and Subiculum area at 1 week after insult. Fluorescence images show several FJB (+) neurons in the CA1, CA3, hilus and subiculum area at 1 week after seizure. Intraperitoneal treatment of clioquinol for 1 week provided not protective effects on hippocampal neuronal death after seizure compared to vehicle (DMSO) treated group. Scale bar = 200 µm. (B) Bar graph shows the quantification of neuronal degeneration in the hippocampus. The number of FJB (+) neurons is not different between vehicle and clioquinol treated group in the CA1, CA3, hilus and subiculum area. *P<0.05.

**Figure 2:**
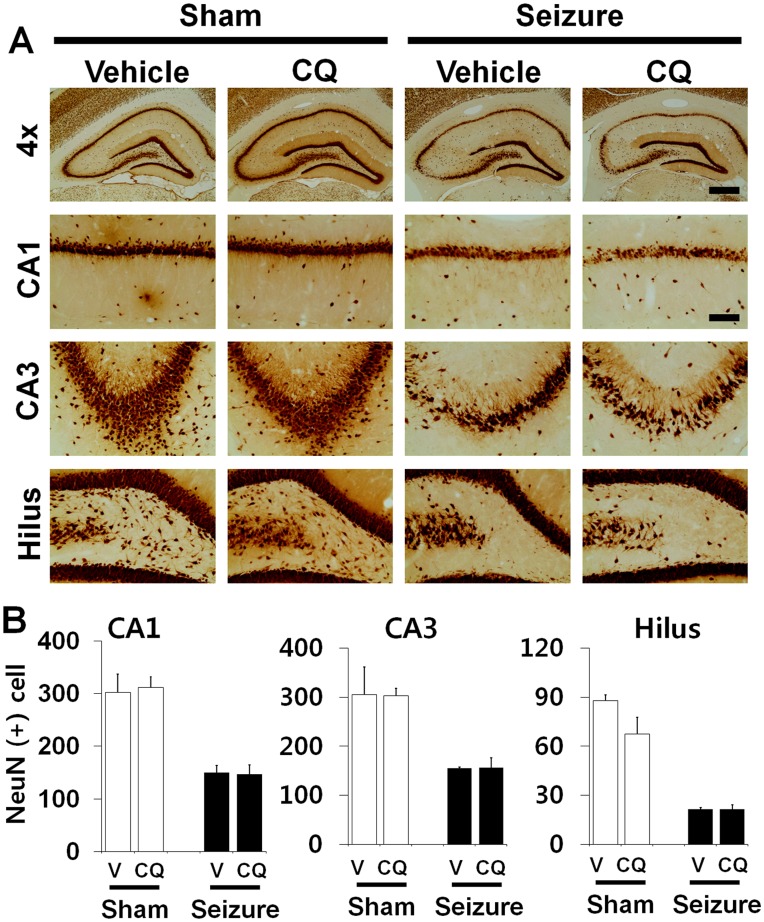
Seizure-induced hippocampal neuronal loss is not prevented by clioquinol. Live neurons after seizure were detected by NeuN staining in the hippocampal CA1, CA3 and hilus regions at 1 week after insult. Light microscopic images show decreased NeuN (+) neurons in the CA1, CA3 and hilus area at 1 week after seizure. Intraperitoneal injection of CQ provided no protective effects on hippocampal neuronal death. Scale bar = 250 µm. (B) Bar graph shows that the number of NeuN (+) neurons is not statistically different between vehicle and CQ treated rats. Data are means ± SE, n = 6 from each group. *P<0.05.

## Materials and Methods

### Ethics Statement

Animal studies were approved by the Committee on Animal Use for Research and Education at Hallym University (protocol # Hallym 2010-64-1), in compliance with NIH guidelines. Animal sacrifice was performed under isoflurane anesthesia, and all efforts were made to minimize suffering.

### Animals Handling

Animals were housed 2 per cage under conditions of constant room temperature 18–20°C and humidity 50–55%, and had free access to tap water and food. Room lights were automatically turned on at 6∶00 and off at 18∶00. In this study, we used 8 week old male Sprague-Dawley rats (250–300 g, DBL Co, Korea). Rats were fed with a normal zinc containing diet (Purina, Gyeonggi, Korea) for the entire experiment.

**Figure 3:**
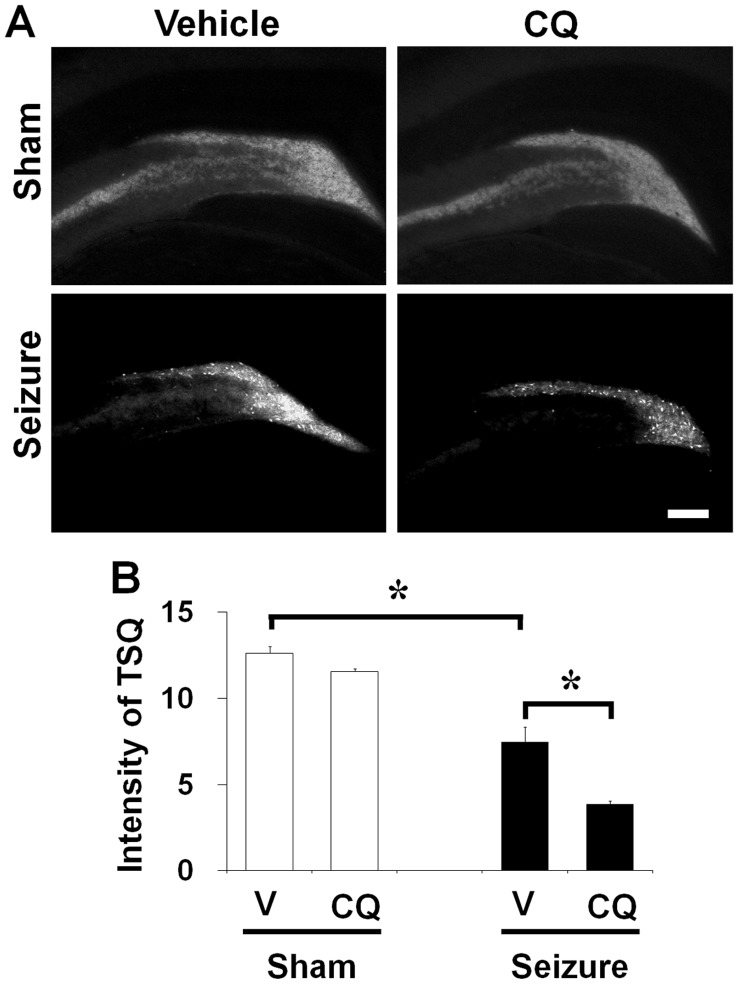
Clioquinol reduced TSQ intensity after seizure. (A) TSQ fluorescence image in the dentate granule cell layer 1 week after sham-operated or seizure-experienced rats. Vesicular TSQ intensity is high in mossy fiber area of dentate granule cell layer in sham operated rats. However, 1 week after seizure the vesicular TSQ fluorescent intensity is decreased in the mossy fiber area. CQ treatment decreased TSQ intensity of mossy fiber area either in sham operated rats or in seizure-experienced rats. Scale bar = 200 µm. (B) A graph represents quantitated intensity of TSQ fluorescent in the hilar area. CQ treated group shows significantly lower TSQ intensity than vehicle treated group in 1 week after seizure (n = 8). Data are means ± SE. *P<0.05.

### Pilocarpine-induced Seizure

To investigate the role of zinc on seizure-induced progenitor cell proliferation, rats underwent a lithium-pilocarpine epilepsy model. Pilocarpine-induced seizure model for rats was performed as described previously [17]. Rats were treated with lithium chloride 19 hours before pilocarpine injection (Sigma-Aldrich Co., St. Louis, MO, 127 mg/kg, i.p.). Pilocarpine (Sigma-Aldrich Co., St. Louis, MO, 25 mg/kg i.p.) was administrated intraperitoneally (i.p.) in the morning. Pretreatment with scopolamine (Sigma-Aldrich Co., St. Louis, MO, 2 mg/kg, i.p.) 30 min prior to pilocarpine injection was used to suppress peripheral cholinergic effects. Status epilepticus (SE) typically occurred within 20–30 min of the pilocarpine administration. Rats were placed in individual observation chambers in which seizure activity (stereotyped oro-facial movements, salivation, eye-blinking, twitching of vibrissae, straub tail, stiffened hindlimbs and reduced responsiveness) were observed. Diazepam (Valium, Hoffman la Roche, Neuilly sur-Seine, France, 10 mg/kg, i.p.) was administered two hours after onset of SE and repeated as needed for seizure termination. Blood glucose was measured with an ACCU CHECK glucose analyzer (ACCU CHECK GO, Co., Hoffman la Roche, Neuilly sur-Seine, France) before and after seizure. Animals were returned to their cages when fully awake and ambulatory.

**Figure 4:**
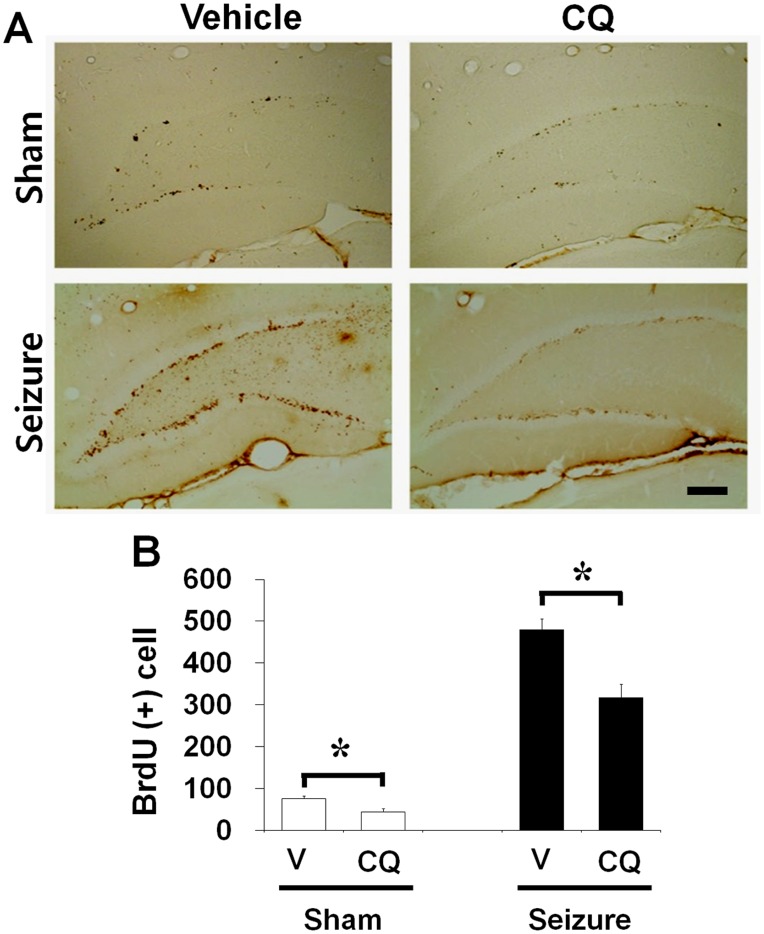
Clioquinol reduced number of BrdU-labeled cells in the dentate gyrus. Bromodeoxyuridine binding cells emerged in the dentate gyrus of rats. (A) Brains were harvested at 1 week after seizure and then brain sections were immunohistochemically stained with BrdU. BrdU (+) cells were reduced by zinc chelation in the dentate gyrus at 1 week after seizure. BrdU (+) cells were significantly higher in seizure-induced rats than in the sham operated rats. Seizure-induced BrdU (+) cell production was reduced by CQ. Scale bar = 200 µm. (B) Bar graph represents BrdU-immunoreactive (+) cell number in the subgranular zone area (n = 8). Data are means ± SE. *P<0.05.

**Figure 5:**
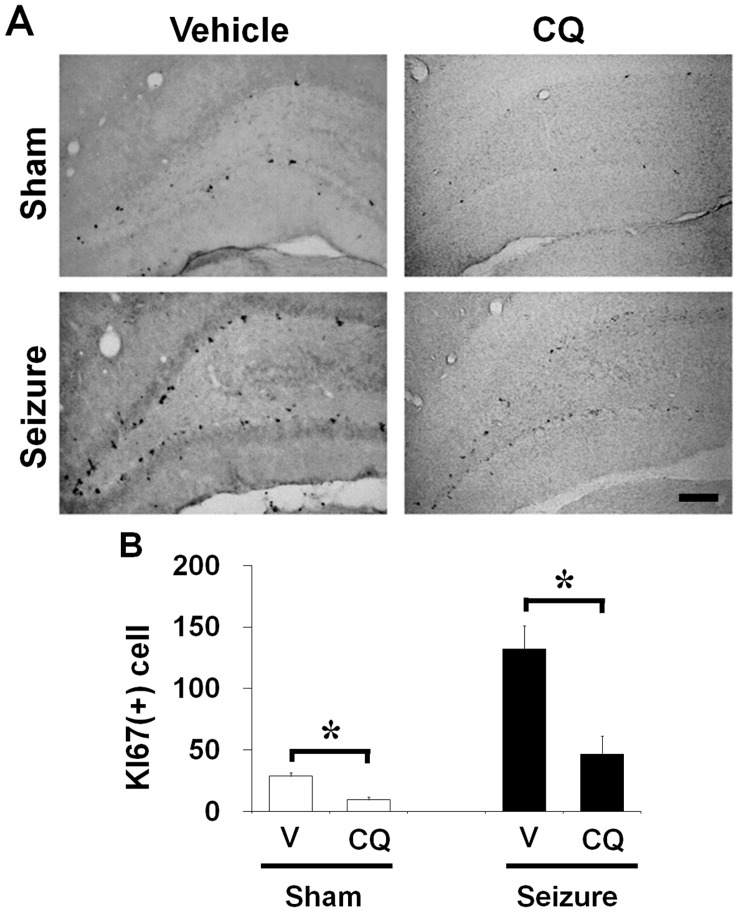
Clioquinol reduced the number of Ki67-labeled cells in the dentate gyrus. Progenitor cell proliferation emerged in the dentate gyrus of rats. (A) Brains were harvested at 1 week after seizure and then brain sections were immunohistochemically stained with Ki67. Progenitor cell proliferation was significantly higher in seizure-induced rats than in the sham operated rats. Seizure-induced progenitor cell proliferation was reduced by CQ. Scale bar = 200 µm. (B) Bar graph represents number of Ki67-immunoreactive cell in the subgranular zone of DG (n = 8). Data are means ± SE. *P<0.05.

### Zinc Chelators Injection

To depress vesicular zinc levels or to chelate extracellular zinc, two zinc chelators, clioquinol and N,N,N0,N-Tetrakis (2-pyridylmethyl) ethylenediamine (TPEN) were used. Eight weeks old male rats were injected with clioquinol (CQ, Sigma, St. Louis, MO, 30 mg/kg, i.p.) and TPEN (5 mg/kg. s.c.) twice per day (9–10 AM and 17–18 PM) for 1 week after pilocarpine-induced seizure or without seizure. Clioquinol was dissolved with dimethyl sulfoxide (DMSO, 30 mg/100 µL, Sigma, St. Louis, MO) and then injected by intraperitoneally (i.p). In the seizure experienced rats, CQ injection was started immediately after 2 hours of epilepsy. Control rats were injected with the same volume of DMSO. The non-seizure group also had CQ/DMSO or DMSO vehicle only. TPEN solution was freshly prepared in 10% ethanol (10% ethyl alcohol in normal physiological saline, Merck, Darmstadt, Germany) and administered under the nape skin of the animals. Rats were treated for seven successive days at doses of 5 mg/kg body weight. As a control, an equivalent volume of 10% ethanol was administered daily for 7 days.

**Figure 6:**
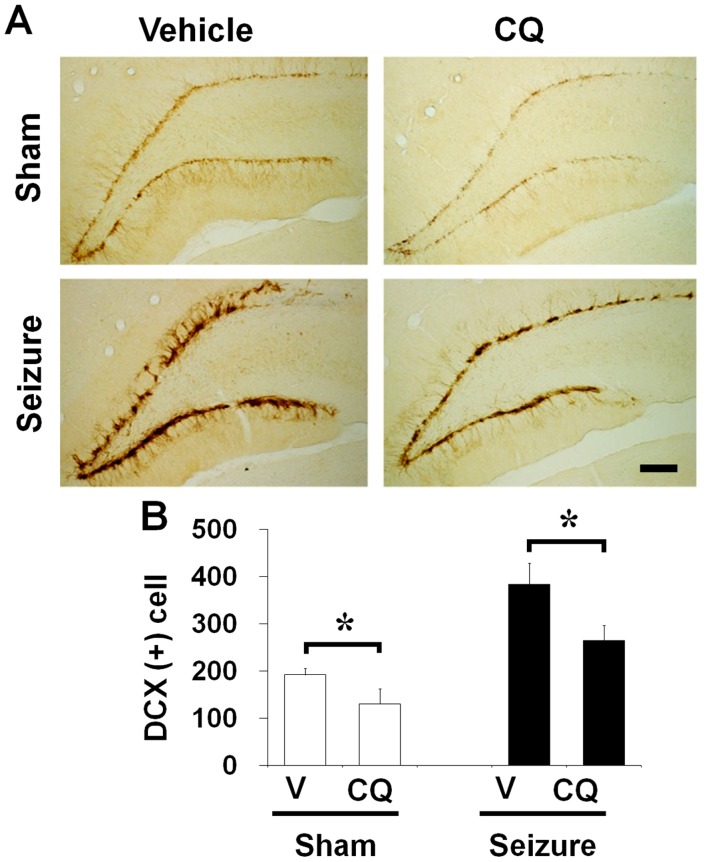
Clioquinol reduced the number of DCX-labeled cells in the dentate gyrus. The neuroblast marker, doublecortin (DCX), is up-regulated in the dentate gyrus of rats after seizure. (A) Brains were harvested at 1 week after seizure and then brain sections were immunohistochemically stained with DCX. DCX (+) cells were significantly higher in seizure-induced rats than in the sham operated rats. DCX was reduced by CQ in the dentate gyrus at 1 week after seizure. In the sham operation, DCX (+) cells were also reduced by CQ. Scale bar = 200 µm. (B) Bar graph represents number of DCX-immunoreactive cell in the subgranular zone of DG (n = 8). Data are means ± SE. *P<0.05.

**Figure 7:**
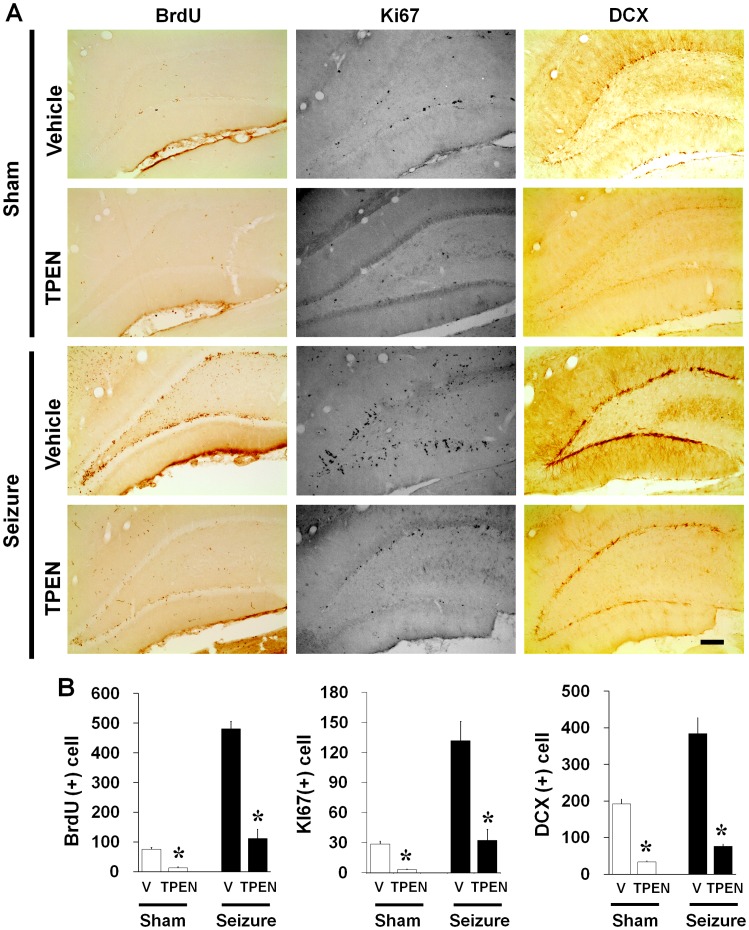
Intracellular zinc chelator, TPEN, reduced the number of newly generated cells in the dentate gyrus. (A) Light microscope images show BrdU (+) cells, Ki67 (+) cells and DCX (+) cells. One week injection of intracellular zinc chelator, TPEN, reduced the number of BrdU (+) cells, Ki67 (+) cells and DCX (+) cells with or without seizure. Scale bar = 200 µm. (B) Bar graph represents number of BrdU, Ki67 and DCX-immunoreactive cell in the subgranular zone of DG (n = 5). Data are means ± SE. *P<0.05.

### Evaluation of Neuron Degeneration

Neuronal death after seizure was evaluated 1 week later. Rats were deeply anesthetized by 5% isoflurane, and all efforts were made to minimize suffering. Rats were intracardially perfused with 0.9% saline followed by 4% paraformaldehyde (PFA). The brains were post-fixed with 4% PFA for 1 hour and then incubated with 30% sucrose for cryoprotection. Brain sections were stained for the Fluoro-Jade B staining (FJB, Histo-Chem Inc., Jefferson, AR) [18], [19]. Degenerating cells were detected with 450_ ¯_490 nm excitation and a 515 nm emission filter. Five coronal sections were collected from each animal by starting 4.0 mm posterior to Bregma, and collecting every ninth section until 5 sections were in hand. These sections were then coded and given to a blinded experimenter who counted the number of degenerating neurons in the hippocampal CA1, cubiculum and hilus.

### Detection of Live Neuron

To identify neuroprotective effects of CQ after seizure, brain sections were immunohistochemical stained with NeuN. Monoclonal anti-NeuN, clone A60 antibody (diluted 1∶100, Millipore, Bellerica, MA) was used as the primary antibody in PBS containing 0.3% Triton X-100 overnight at 4°C. The sections were washed three times for 10 min with PBS, incubated in biotinylated anti-mouse IgG (Vector, Burlingame, CA) and ABC complex (Vector, Burlingame, CA), diluted 1∶250 in the same solution as the primary antiserum. Between the incubations, the tissues were washed with PBS three times for 10 min each. The immune reaction was visualized with 3,3 = -diaminobenzidine (DAB, Sigma-Aldrich Co., St. Louis, MO) in 0.01 M PBS and mounted on the gelatin-coated slides. The immunoreactions were observed under the Axioscope microscope (Carl Zeiss, Munchen-Hallbergmoos, Germany).

### Fluorescence Zn^2+^ Staining (TSQ Method)

Vesicular free zinc was imaged using the N-(6-methoxy-8-quinolyl)-para-toluenesulfonamide (TSQ) method [20]. Rats were euthanized 3 h after CQ (30 mg/kg) treatment and the fresh frozen brains were coronally sectioned. Five evenly-spaced sections were collected through the hippocampal region of each brain and immersed in a solution of 4.5 µmol/L TSQ (Molecular Probes, Eugene, OR) for 60 seconds, then rinsed for 60 seconds in 0.9% saline. TSQ-zinc binding was imaged and photographed with a fluorescence microscope with 360 nm UV light and a 500 nm long-pass filter. The mean fluorescence intensity within the mossy fiber terminal area was measured and expressed as arbitrary intensity after subtraction of background fluorescence as measured in the lateral ventricle. Measurements from the five sections were averaged for each observation.

### BrdU Labeling

To test the effects of zinc chelation on neurogenesis, BrdU was injected twice daily for four consecutive days starting 3 days after the seizure. The thymidine analog BrdU was administered intraperitoneally (50 mg/kg; Sigma, St. Louis, MO) to investigate the progenitor cell proliferation. The rats were killed 7 days after seizure. To test the zinc chelation effects on neurogenesis after seizure, rats received twice daily injections of BrdU for four consecutive days from the 3rd day following seizure and killed on day 7.

### Immunohistochemistry Staining

Rats were anesthetized with urethane and then transcardially perfused by 4% paraformaldehyde (PFA) in 0.1M phosphate buffer (PB, pH 7.4). The brains were removed post-fixed for 1 hour, and then cryoprotected by 30% sucrose. 30-µm free floating coronal sections were immunostained as described [4] using the following reagents: mouse anti-BrdU (Roche, Indianapolis, IN); rabbit anti-Ki67 (recognizing nuclear antigen expressed during all proliferative stages of the cell cycle except G_0_ [21], Novocastra, UK); guinea pig anti- doublecortin (DCX) (recognizing immature neurons [22], Santa Cruz Biotechnology, CA), ABC solution (Vector laboratories, Burlingame, CA).

### Cell Counting

For BrdU, Ki67 and DCX Immunohistochemistry, every ninth coronal section spanning the septal hippocampus was collected. Five coronal sections were collected from each animal by starting 4.0 mm posterior to Bregma, and collecting every ninth section until 5 sections were in hand. These sections were then coded and given to a blinded experimenter who counted the number of BrdU, Ki67 and DCX -immunopositive cells in the SGZ and granule cell layer (GCL).

### Statistical Analysis

All data were expressed as means ± SE. The statistical significance of differences between means was calculated using SPSS (SPSS Inc, Chicago, IL). For statistical comparisons between data from normal and from zinc chelator treated rats in BrdU, Ki67 and DCX positive cells, significance was determined using one-way ANOVA followed by Bonferroni post hoc test. For statistical comparisons between data from all other experiments, significance was evaluated by two-tailed Student t-test. *P* values <0.05 were considered significant.

## Results

### Seizure-induced Hippocampal Neuronal Death is not Prevented by CQ

To test whether CQ treatment shows neuroprotective effects after pilocarpine-induced seizure, rats were sacrificed 1 week after insult with or without CQ injection. Neuronal injury was evaluated by FJB staining. Widespread FJB (+) neurons were detected in the hippocampal CA1, CA3, hilus and subiculum area in the vehicle treated rats 1 week after seizure (Fig. 1). Surviving neurons were evaluated by NeuN staining 1 week after seizure. NeuN (+) neurons disappeared in the hippocampal CA1, CA3 and hilus in vehicle treated rats at this time point. Compared with vehicle-treated rats, CQ-treated rat showed a similar number of NeuN (+) neurons in the hippocampal CA1, CA3, hilus and subiculum area, suggesting neuronal death is not prevented by CQ (Fig. 2).

### CQ Decreased Hippocampal Vesicular Zinc Level in Normal or after Pilocarpine-induced Seizure

To test whether CQ treatment decreases vesicular zinc intensity in the mossy fiber of hippocampus, brain sections were stained by TSQ. Consistent with previous observations [5], [23], the intensity of mossy fiber zinc in the rat hippocampus was 48.3% lower in CQ treatment group than in vehicle treated controls (Fig. 3).

### Progenitor Cell Proliferation in the Subgranular Zone of Dentate Gyrus is Reduced by CQ in Normal and Post-seizure Subjects

To test whether CQ affects progenitor cell proliferation in the adult brain, rats were sacrificed 1 week after continuous CQ treatment without seizure. Rats were injected with BrdU twice per day for 4 days in both vehicle or CQ treated group. Cell proliferation was assessed by Ki67 and BrdU immunohistochemistry. We found decreased number of Ki67 and BrdU labeled cells in rats without seizure (Fig. 4). To investigate how CQ affected seizure-induced progenitor cell proliferation and neurogenesis, rats were injected with BrdU twice per day from 4 days after pilocarpine-induced seizure until to sacrifice. Rats were injected with CQ from 2 hours after seizure twice per day for 1 week. Cell proliferation was assessed by Ki67 and BrdU immunohistochemistry. We observed increase in the number of cells labeled by both Ki67 and BrdU staining in rats that underwent pilocarpine-induced seizure at 1 week after seizure compared to sham operation. However, a group of 1 week CQ treated rats showed lower number of Ki67 and BrdU immunoreactive cells in the DG of hippocampus after seizure compared to vehicle treated group (Fig. 5).

### Neuroblast Production in the Subgranular Zone of Dentate Gyrus is Reduced by CQ in Normal and Post-seizure Subjects

To investigate how CQ affects neuroblast migration, normal or seizure-experienced rats were continuously injected with CQ. Doublecortin (DCX) is a microtubule-associated protein expressed by immature neurons. The levels of DCX expression increase in response to seizure, which occurs in parallel with BrdU labeling in measuring neurogenesis. In normal rats, CQ or vehicle was injected into the intraperitoneal space twice per day for 1 week. In seizure experienced rats, CQ or vehicle was injected at 2 hours after seizure, and then the CQ injection was continued twice per day for 1 week. Number of neuroblast was assessed by DCX immunohistochemistry. In the normal rats (without seizure), the number of DCX stained neurons in DG area is lower in CQ injected group than vehicle treated group. The number of DCX immunoreactive cells is significantly increased at 1 week after seizure compared to sham operated animals. However, CQ treated rats showed lower number of DCX immunoreactive cells in the DG of hippocampus compared to vehicle treated group after seizure (Fig. 6).

### Progenitor Cell and Neuroblast Proliferation in the Subgranular Zone of Dentate Gyrus is Reduced by TPEN in Normal and Post-seizure Subjects

To test whether another zinc chelator, TPEN, also affects progenitor cell and neuroblast proliferation in the adult brain, rats were sacrificed 1 week after continuous TPEN treatment with or without seizure. We found that group of 1 week TPEN treated rats also showed lower number of BrdU, Ki67 and DCX immunoreactive cells in the DG of hippocampus with or without seizure compared to vehicle treated group (Fig. 7).

## Discussion

The present study tested the hypothesis that brain zinc might play a modulatory role in hippocampal neurogenesis either in normal or in epilepsy-experienced rats. This study found that pharmacological zinc chelation substantially reduced basal or seizure-induced progenitor cell proliferation. The present study suggests that vesicular zinc is an important mediator of neuronal regeneration in the hippocampus either under normal physiologic conditions or following brain insult.

Chelatable zinc is highly concentrated in the mossy fiber of dentate granule cell of the hippocampus [24], [25]. Excessive zinc translocation into postsynaptic neurons contributes to neuronal death in several disease conditions, such as prolonged seizures [26], [27], ischemia [28], [29], brain trauma [30], [31] and hypoglycemia [32], [33]. However, an equally abundant number of studies have shown that zinc has many beneficial or constitutive roles in the brain as well [15]. Zinc participates in the regulation of cell proliferation in several ways; it is essential to enzymatic functions that influence cell division and proliferation. Additionally, several studies have shown that zinc deficiency alters postnatal brain development [34]. Thus, the evidence outlined above confirms that zinc is an essential transition element in cell division and proliferation, and further suggests that zinc has a critical role in neurogenesis in the developing brain.

The dentate granule (DG) cells have the unique property of prolonged postnatal neurogenesis within the hippocampal formation [35], [36] [37]. Hippocampal neurogenesis is continued through adulthood in the rodents [38], [39], [40], [41]. Neuronal precursor cells reside in the SGZ of the dentate gyrus, where they proliferate continuously into the granule cell layer [41], [42], [43]. The precursor cells eventually develop granule cell morphology and begin to express markers of differentiated neurons [43], [44]. In addition to lifelong physiological neurogenic properties, dentate granule cells are believe to be involved in the pathogenesis of temporal lobe epilepsy, one of the most common human seizure disorders [45], [46], [47]. After seizure, the dentate granule cells produce abnormal axonal projections to the supragranular inner molecular layer of the dentate gyrus. This unique process after epilepsy, called “mossy fiber sprouting”, can be identified by Timm staining of zinc [48]. Mossy fiber sprouting may result in recurrent excitatory circuits or stabilize the network by innervating inhibitory neurons. Dentate granule cell neurogenesis and seizure-induced hippocampal network reorganization in adult rodent raises the possibility of a relationship between these two phenomena. Given the data on continuing granule cell neurogenesis, Parent et al. showed that hippocampal plasticity associated with recurrent seizures is derived primarily from newly born granule cells rather than from existing and mature dentate granule cells [2], [3].

To test the hypothesis that zinc is essential for neurogenesis, we used the chemical zinc chelator, CQ, to directly test the zinc deprivation effects on hippocampal neurogenesis. Our previous study described a transient increase of progenitor cells after hypoglycemia until 2 weeks after insult [4]. The reason for an increase in neurogenic activity at early time points after hypoglycemia is uncertain. Thus, we speculated that this transient increase of neurogenesis after seizure is related to synaptic release of zinc and cytolysis after dentate granule cell degeneration. Our present study demonstrates several zinc accumulating neurons in the dentate granule cell and hilar cell bodies after seizure. Previously we suggested that those zinc-accumulated neurons were degenerating after seizure [27]. We believe that continuous liberation of free zinc from the degenerating dentate granule cells or from mossy fiber synaptic terminals may chronically stimulate progenitor cell proliferation and support survival of neuroblast after hypoglycemia insult. Therefore, we tested the effects of zinc chelation on basal neurogenesis as well as on seizure-induced transient neurogenesis. Continuous treatment with CQ for 1 week without seizure significantly decreased basal progenitor cell proliferation in the hippocampus compared to the vehicle treated group, with a parallel reduction in the number of neuroblasts. Moreover, 1 week of continuous treatment with CQ after seizure also substantially reduced progenitor cell proliferation in the hippocampus. These results suggest that zinc in the brain modulates neurogenesis after epilepsy. However, a major concern regarding the use of CQ is that this chelator is not entirely zinc specific, since CQ also can chelate other transitional metals in the brain such as copper and iron [23]. To verify our present finding that reduction of neurogenesis by CQ treatment is solely due to depletion of extracellular zinc we will need a more specific zinc chelator for the future study. Another concern is that CQ may not only act as a zinc chelator but also act as a zinc ionophore [49]. However, we speculate that CQ binds with chelatable (or free) zinc in the extracellular space and in the intracellular area, which depresses brain zinc availability to support neurogenesis either in the basal setting or after seizure. To differentiate whether zinc chelation or zinc ionophore effect of CQ may cause counter neurogenesis alternatively we delivered N,N,N′,N′-tetrakis(2-pyridylmethyl)ethylenediamine (TPEN) after seizure for 1 week. In the present study, we found that intracellular zinc chelator, TPEN, also significantly reduced seizure-induced neurogenesis. This finding is consistent with previous published study using cultured human neuronal precursor cells that TPEN treatment resulted in significant decrease in cellular proliferation [50]. Thus, these data suggest that zinc plays a role in neurogenesis and zinc chelation reduces brain injury-induced neurogenesis.

Taken together, our present study demonstrates that vesicular zinc in the hippocampus modulates neurogenesis in the adult brain under physiological as well as pathological conditions. Elucidation of the mechanisms involved in the zinc-mediated hippocampal neurogenesis warrant further investigations.
